# Epidemiological Survey of Peste des Petits Ruminants in Ethiopia: Cattle as Potential Sentinel for Surveillance

**DOI:** 10.3389/fvets.2019.00302

**Published:** 2019-09-12

**Authors:** Getahun E. Agga, Didier Raboisson, Ludovic Walch, Fitsum Alemayehu, Dawit T. Semu, Getahun Bahiru, Yilkal A. Woube, Kelay Belihu, Berhe G. Tekola, Merga Bekana, François L. Roger, Agnès Waret-Szkuta

**Affiliations:** ^1^Food Animal Environmental Systems Research Unit, Agricultural Research Service, United States Department of Agriculture, Bowling Green, KY, United States; ^2^IHAP, INRA, ENVT, Université de Toulouse, Toulouse, France; ^3^College of Veterinary Medicine and Agriculture, Addis Ababa University, Bishoftu, Ethiopia; ^4^Department of Biomedical Sciences, College of Veterinary Medicine, Tuskegee University, Tuskegee, AL, United States; ^5^Food and Agriculture Organization of the United Nations, Addis Ababa, Ethiopia; ^6^Food and Agriculture Organization of the United Nations, Rome, Italy; ^7^CIRAD, UMR ASTRE, Montpellier, France

**Keywords:** peste des petits ruminants, cattle, sheep, goats, agroecology, seroprevalence

## Abstract

Peste des petits ruminants (PPR) is a highly contagious viral disease of small ruminants; it emerged in countries previously free of the disease following the eradication of rinderpest. PPR is classified by international organizations as the next priority animal disease for global eradication campaign. Assessment of the local situations is the first step in the eradication efforts. The objective of this study was to investigate and compare the seroprevalence of PPR in cattle, sheep, and goats under two livestock production systems in Ethiopia: North Shewa zone of Amhara region represents a highland sedentary life style characterized by mixed livestock-crop production system; Zone Three of Afar region represents a lowland nomadic life style characterized by pastoral livestock production system. N-competitive ELISA PPR test was performed on sera from 2,993 animals ≥6 months old sampled at watering and grazing points. Multivariable logistic regression models comparing the seropositivity between the two production systems were built by classifying doubtful results as positive, negative, or excluding them from the data. The odds ratio (OR) comparing overall PPR seroprevalence in the sedentary North Shewa Zone compared to the nomadic Zone Three ranged from 19 to 27 (*P* < 0.001), depending on how doubtful results were classified, which contrasts with what has been reported in the literature. This is not likely to be related solely to vaccination, since seroprevalences in cattle and small ruminants were similarly high or low in the respective zones (0–4% for Zone Three and 20–40% for North Shewa Zone), and cattle were not likely to be vaccinated. The OR of seropositivity for goats compared to cattle ranged from 1.9 [95% confidence interval (CI): 1.3–2.7; *P* < 0.001] to 2.2 (95% CI: 1.5–3.1; *P* < 0.001) when doubtful results were excluded or classified as negative, respectively. When doubtful results were classified as positive, association between seropositivity and animal species was not significant (*P* > 0.05). Our results suggest to further investigate cattle as sentinel animals for PPR surveillance.

## Introduction

Peste des petits ruminants (PPR) is a highly contagious viral disease of small ruminants. Since it was first identified in Ivory Coast in 1942, its geographic distribution has been expanding within Africa, and spread to the Middle East and Asia (1). PPR gained international attention following the detection of PPR virus in Turkey in 1996 with the fear that the disease can spread to the rest of Europe and other developed countries (1). PPR is the next priority animal disease targeted for global eradication campaign by Food and Agricultural Organization of the United Nations (FAO) and the World Organization for Animal Health (OIE) (2, 3). The disease is characterized clinically by high fever, pneumonia, necrotic lesions of the oral cavity, and diarrhea; and epidemiologically by high morbidity and mortality rates in small ruminants (4, 5). Although cattle, swine, camels, and buffaloes can be infected with the PPR virus (6–8), the role of these species in the epidemiology of the disease is still unclear (1, 4).

In Ethiopia, the first clinically suspected case of PPR was reported from goat herds in Afar region in 1977 and later was confirmed through the isolation of the virus in 1991 (9, 10). Since then, PPR has been reported from various parts of the country with seroprevalences varying between 12% in 2001 similarly to that of the national serological survey conducted in 1999 and 31% in 2009–2010 in pastoral flocks (11–14). The disease is considered endemic in the country and control relies solely on immunization of small ruminants as an efficacious live attenuated vaccine producing lifelong immunity against all PPR virus serotypes after a single administration is available (15). The strategy is mass vaccination in lowlands and ring vaccination following PPR outbreaks in the highlands considering the different production systems in the two agro-ecological zones. Animal movements in the lowlands are more frequent and commonly involve large number of animals which puts them at a higher risk for PPR infection. In addition, vaccination fees may not be affordable in the pastoral communities (15, 16). The objective of this study was to investigate and compare seroprevalences of PPR in cattle, sheep, and goats in two different but contiguous zones of Ethiopia representing on one hand a highland sedentary livestock farming system (North Shewa Zone in Amhara Region) and on the other hand a lowland pastoral nomadic system (Zone Three in Afar Region) and discuss the implication of the findings for the design of surveillance and control activities.

## Materials and Methods

**Figure 1:**
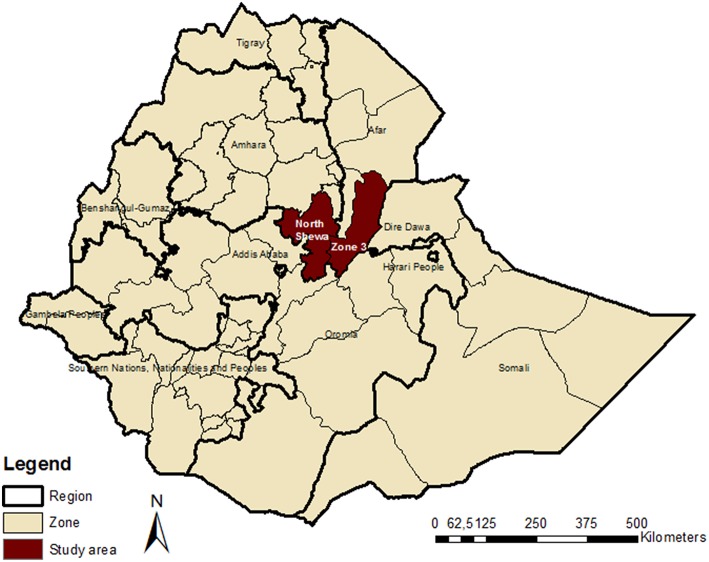
Map of Ethiopia showing the study Zones for serological survey of Peste des petits ruminants in cattle and small ruminants.

A cross-sectional study was carried out from December 2005 to June 2006. Blood samples were collected from as many as possible number of cattle, sheep, and goats in two different agroecological zones. North Shewa Zone in the Amhara Region is situated in the highlands (>1,200 m above sea level) of Ethiopia where mixed livestock and crop production prevails; Zone Three of Afar Region is in the lowlands and is characterized by pastoral nomadic husbandry system (Figure 1). Farmers were asked for their consent to participate in the study at watering and grazing points and were purposively selected because of logistics for field sampling and time constraints. In the affirmative, animals believed to be over 6 months old in the herd were sampled, to avoid seropositivity due to maternal antibodies. Blood samples were collected from the jugular vein into plain vacutainer tubes and were kept overnight at room temperature to clot. Serum was separated from the clot by simple decantation or by centrifugation when necessary. Sera were transferred into cryovials and kept at −20°C until analyzed in the laboratory.

Serum samples were tested for the presence of specific PPR antibodies by using N-competitive enzyme linked immunosorbent assay (N-cELISA) kit according to the manufacturer's instructions (CIRAD/EMVT, Montpellier, France), at National Veterinary Institute (Bishoftu, Ethiopia). The cELISA kit was based on recombinant N-protein of PPR virus as the capture antigen and a monoclonal antibody against the N-protein as the competitive antibody (17). The optical densities (OD) were measured with an ELISA reader with an inference filter of 492 nm. The percent inhibition (PI) values were determined according to the following formula:

PI (%) = 100−[OD of control or test serum/OD of monoclonal control]^*^100. The PI values were categorized as negative (PI < 45%), doubtful (PI = 45–49%) or positive (PI ≥ 50%).

Multivariable logistic regression models for the outcome seropositive for PPR (0 or 1) were run separately by classifying doubtful results as (i) negative, (ii) positive, or (iii) excluded, and including the explanatory variables Zone and species. Two-way interactions between the variables were tested. Statistical analyses were performed with R version 3.2.3 using the glm function (18). Results were considered statistically significant when *P* < 0.05.

## Results

The proportion of positive results by species and by Zone when doubtful laboratory results were classified as positive, negative, or excluded is presented in Table 1. The odds of seropositivity for the combined results of the three animal species in North Shewa Zone compared to Zone Three ranged from 19 to 27 (Table 1), for the three scenarios of how doubtful results were classified. The odds of seropositivity for goats compared to cattle was 2.2 [95% confidence interval (CI): 1.5–3.1], 1.3 (95% CI: 0.9–1.8), and 1.9 (95% CI: 1.3–2.7) when doubtful results were classified as negative, positive, or excluded, respectively. Zone by animal species interaction (data not shown) was not statistically significant (*P* > 0.05) in the three scenarios considered for the doubtful values; results reported here therefore represent the main effects of zone and animal species adjusted for the effect of the other in the multivariable logistic regression models.

**Table 1:** Descriptive and logistic regression analyses results comparing seropositivity (dichotomous outcome recorded as seropositive or seronegative) of peste des petits ruminants between sedentary highland (North Shewa Zone, Amhara region) and nomadic lowland (Zone Three, Afar region) livestock production systems in Ethiopia, December 2005–June 2006.

**Outcome classification and variables**			**No. animals tested**	**Seroprevalence (%)**	**Odds ratio (OR)**		
					**OR**	95% CI*	***P*-value**
≪Doubtful≫ classified as negative	Zone	Zone Three	1,953	2.1			
		North Shewa Zone	1,040	25.7	19.4	13.8–27.9	<0.001
	Species	Cattle	6,13	10.6			
		Goats	1,325	9.6	2.2	1.5–3.1	<0.001
		Sheep	1,055	11.0	1.3	0.9–1.9	0.085
≪Doubtful≫ classified as positive	Zone	Zone Three	1,953	2.4			
		North Shewa Zone	1,040	38.2	27.1	19.7–37.9	<0.001
	Species	Cattle	613	18.6			
		Goats	1,325	10.9	1.3	0.9–1.8	0.062
		Sheep	1,055	17.4	1.2	0.9–1.6	0.146
≪Doubtful≫ excluded	Zone	Zone Three	1,948	2.1			
		North Shewa Zone	910	29.3	22.2	15.8–31.9	<0.001
	Species	Cattle	564	11.5			
		Goats	1,307	9.7	1.9	1.3–2.7	<0.001
		Sheep	987	11.7	1.3	0.9–1.9	0.082

*Results are presented by classifying doubtful laboratory results as positive, negative, or excluding them. Results were considered significant when P < 0.05*.

* *95% confidence interval for the odds ratio (OR)*.

The proportions of seropositive and doubtful results in the two zones and the three livestock species is presented in Table 2. The proportions of laboratory doubtful results for cattle and sheep (15.5% each) were considerably higher in North Shewa Zone compared to Zone Three (0.0% in cattle and 0.2% in sheep) (Table 2). Similarly, the proportions of test positive results for cattle and sheep (20 and 22%, respectively) were much higher in the North Shewa Zone than in Zone Three (0.3% in cattle and 0.5% in sheep). For goats the doubtful results were relatively considerably lower (4.8% in the North Shewa Zone and 0.4% in Zone Three) than that of sheep and cattle in both zones. However, we note that in both zones the highest seroprevalence (considering the proportion of test classified positive results) was observed in goats (3.6% in Zone Three and 31% in North Shewa Zone).

**Table 2:** Percentage of animals tested doubtful or positive for peste des petits ruminants in Zone Three of Afar region and North Shewa Zone of Amhara region, Ethiopia, December 2005–June 2006.

**Zone**	**Species**	**Number of animals tested**	****%** Doubtful**	****%** Positive**
Zone Three	Cattle	296	0.0	0.3
	Goats	1,035	0.4	3.6
	Sheep	622	0.2	0.5
	Zone Three total	1,953	0.3	2.1
North Shewa Zone	Cattle	317	15.5	20.2
	Goats	290	4.8	31.0
	Sheep	433	15.5	21.6
	North Shewa Zone total	1,040	12.5	25.7
Species total	Cattle	613	8.0	10.6
	Goats	1,325	1.4	9.6
	Sheep	1,055	6.5	11.0
	Overall total	2,993	4.5	10.3

## Discussion

Similar seroprevalences for cattle and small ruminants have been found in some other studies (7, 11). This can be explained by the fact that mixed herds of different animal species likely transmit PPR virus to contact animals. Since the turn-over rate of cattle is lower (10%) than that of small ruminants (30%) (particularly in goats), and since goats are more likely to succumb to PPR disease than sheep and cattle the seroprevalence rates in sheep and cattle may occasionally be higher than that of goats (2, 19) as observed in the present study.

The results showed a significantly higher PPR seroprevalence in the sedentary highland North Shewa Zone compared to the lowland pastoral nomadic Zone Three. This is in contrast with what has been reported in the literature where lowland pastoral nomadic practices have been associated with higher PPR seroprevalence due to large number of animals in continuous movement in search of fodder and water, whereas animal mixing is less frequent in the highlands with small sedentary herds (14, 20). However, more recently, Fentie et al. (21) reported that small ruminants reared in the lowland and highland areas were more affected than those reared in midland (25, 14.58 vs. 7.5% respectively, *P* < 0.05). The difference between the present results and literature may be due to different sampling procedures in the different studies that affect their representativeness. Field collection of data and the use of probability sampling designs are challenging in Ethiopia because of poor infrastructure, cultural differences that may result in a lack of co-operation from livestock owners and periods of hot climatic conditions (22). The field data from our study did not allow a detailed evaluation of the role of the herd size, species composition of the herds and the production system (sedentary highland and lowland pastoral nomadic) on the seroprevalence rates in the two regions which may also explain the difference between results of our study and the literature. Seasonality of the disease also might have affected the results as the time period of the study was limited and outbreaks are more frequent during the main rainy season which typically lasts from March to October in Ethiopia (21). Thus, presence of active PPR outbreaks at the time of serum sample collection in one or both zones studied also could have affected our results. However, samples were obtained from apparently healthy animals and there was no indication of PPR outbreak during the field sampling. It may also be due to differences in prior vaccination status of the animals. The higher seropositivity observed in the highland zone in the present work can reflect a higher prior vaccination rate in the zone that are generally more accessible as several mass vaccination campaigns have occurred in Ethiopia between 2005 and 2011 (16). Indeed, the cELISA test used cannot differentiate infected and vaccinated animals. However, because cattle are not likely to be vaccinated, and because the proportion of seropositive animals is higher in North Shewa Zone in the three animal species (Tables 1, 2), the difference in the seropositive proportion between the two zones is not likely to be due solely to vaccination and may rather result from natural infection. The higher prevalence in the highland zone may also indicate the expansion of the disease into parts of the country previously free of the disease.

The N-cELISA test used is reported to be a highly specific and sensitive test when compared to virus neutralization test (17) but exact corresponding performances and cut-off values have not been published. Couacy-Hymann et al. (19) using N-cELISA considered PI ≥ 50% as positive and PI > 65% as “high percentage of inhibition” when cattle were experimentally infected with virulent PPR virus strains and showed seroconversion. If the threshold of 50% appears reasonable to consider in the field for interpretation of positive or negative results, we looked at the effect of classifying the laboratory results considered doubtful as positive, negative, or excluding them, as it is the way they are recorded by the laboratory and that no clear instructions have been published to date. When laboratory doubtful results were classified as negative (OR = 2.2) or excluded (OR = 1.9), goats were twice more likely to be seropositive than cattle. Although not significant (OR = 1.3; *P* = 0.062), the same trend was observed in the model when doubtful results were classified as positive. Despite significantly higher odds of seropositivity in goats when doubtful results were classified as negative or excluded, crude seroprevalences were similar for goats (9.6–9.7%) and cattle (10.6–11.5%). On the other hand, considerable differences were found between cattle (18.6%) and goats (10.9%) when doubtful results were classified as positive although statistical analysis did not reveal significant association (Table 1). This might be due to stronger effect of zone than the animal species on the seroprevalence (OR = 27) when doubtful results were classified as positive compared to OR ranging from 19 to 22 when the doubtful results were classified as negative or excluded. Nevertheless, the highest seroprevalence in goats in both zones (31% in North Shewa vs. 3.6% in Zone Three; Table 2) is consistent with the fact that goats are maintenance hosts for the disease whereas cattle appear to be dead end hosts in the epidemiological cycle (19, 23).

Despite differences in the odds of seropositivity between goats and cattle, the difference in the prevalence between the two Zones (the three species combined) suggests that cattle may be used as sentinel animals for surveillance purposes particularly in areas at higher risk for introduction of PPR. The use of cattle as sentinel is also recently suggested by others (23, 24), and is consistent with the fact that cattle are usually considered dead-end hosts for PPR and not normally vaccinated against PPR. Detection of PPR antibodies in the cattle may indicate the exposure of cattle to infected small ruminants during housing, grazing, and watering which is typical of small holder livestock production system in Ethiopia (11).

The fact that 15.5% of cattle and sheep sampled from North Shewa zone were classified as doubtful compared to 4.8% in goats cannot be explained by any differential performance of N-cELISA that may be present in different animal species. The test was first developed and validated using goats and cattle sera at 94.5% sensitivity and 99.4% specificity by using virus neutralization test as a gold standard test (17). A recent study, Bodjo et al. (25) similarly reported 96.4% sensitivity and 97.1% specificity for sera obtained from sheep and goats using virus neutralization as a gold standard. However, any species difference in the test performance, if exists, does not diminish the utility of testing cattle as an indicator of PPR virus circulation or seroprevalence in small ruminant herds. However, under very low prevalence, as observed in Zone Three, results should be interpreted with caution. The fact that 15.5% of sheep from the North Shewa zone were classified as doubtful, similarly to cattle, remains unexplained and needs further investigation particularly cELISA test outputs need to be revised. Since the seropositive results in cattle and sheep were also higher in the North Shewa Zone (20.2–21.6%) than in Zone Three (0.3–0.5%), it is also more likely that the doubtful results would be similarly higher in the North Shewa Zone compared to the Zone Three. So, it is unlikely that the higher percentage of doubtful results is due to higher percentage of doubtful results in the zone but more likely due to the generally higher seroprevalence in the North Shewa Zone which would result in higher positive percentage and therefore also more doubtful results. We speculate the shifting of PPR occurrence toward the highland zone since doubtful and test positive results were higher in the North Shewa Zone compared to Zone Three. It may also be due to the non-random sampling of the study animals performed in the present work due to practical constraints. We also note that the doubtful results were not re-tested or verified by other methods.

Many heterogeneities in the population structure and husbandry practices in Ethiopia could not be captured in this study. The limitations are partly due to lack of variables to be included in the analysis and if new studies will be performed in the future, efforts should be made to include at least timing of successive vaccination campaigns and age of the animals to be sampled as already mentioned by Fournié et al. (26). Results of seropositivity are probably influenced by non-probability sampling method used but, in our opinion, this does not affect the finding that cattle can be used as potential sentinels for the serosurveillance of PPR.

## Conclusion

The present work reports an unexpectedly higher PPR seroprevalence in the sedentary highland North Shewa Zone compared to the lowlands pastoral nomadic Zone Three. Goats were twice as likely to be seropositive compared to cattle. Our results suggest that cattle can be used as sentinel species for PPR surveillance in cattle-small ruminant mixed farming areas, and to monitor the impacts of interventions and disease freedom in high risk areas. This is very important since FAO and the Ethiopian Ministry of Livestock and Fisheries reaffirmed their commitments to eradicate PPR from Ethiopia by 2027. No DIVA vaccine is available to date that can help differentiate infected and vaccinated animals.

## Data Availability

The raw data supporting the conclusions of this manuscript will be made available by the authors, without undue reservation, to any qualified researcher.

## Ethics Statement

According to EU law (Directive 2010/63/UE), procedures which use farm animals according to common veterinary practice, can be done without minister permission and without an ethics committee opinion.

## Author Contributions

GA, FA, DS, GB, YW, KB, BT, MB, and FR conceived and designed the study. FA, DS, and GB collected and analyzed samples. GA, YW, KB, BT, and MB supervised data acquisition. GA, LW, DR, and AW-S organized the dataset and performed statistical analysis. GA wrote the first draft of the manuscript. DR, FR, and AW-S wrote sections of the manuscript. All authors helped in the interpretation of the data, revised the manuscript, read, and approved the final version.

### Conflict of Interest Statement

The authors declare that the research was conducted in the absence of any commercial or financial relationships that could be construed as a potential conflict of interest.
